# Bloomsbury report on mouse embryo phenotyping: recommendations from the IMPC workshop on embryonic lethal screening

**DOI:** 10.1242/dmm.012898

**Published:** 2013-07

**Authors:** David Adams, Richard Baldock, Shoumo Bhattacharya, Andrew J. Copp, Mary Dickinson, Nicholas D. E. Greene, Mark Henkelman, Monica Justice, Timothy Mohun, Stephen A. Murray, Erwin Pauws, Michael Raess, Janet Rossant, Tom Weaver, David West

There was an error published in *Dis. Model. Mech.***6**, 571–579.

The Funding section should read: This study was supported by the European Commission (InfraCoMP – grant no. 284501 – to INFRAFRONTIER) and by the UK Medical Research Council.

The authors apologise for this mistake.

